# Efficient detection of symptomatic and asymptomatic patient samples for *Babesia microti* and *Borrelia burgdorferi* infection by multiplex qPCR

**DOI:** 10.1371/journal.pone.0196748

**Published:** 2018-05-10

**Authors:** Shekerah Primus, Lavoisier Akoolo, Samantha Schlachter, Kristine Gedroic, Albert D. Rojtman, Nikhat Parveen

**Affiliations:** 1 Department of Microbiology, Biochemistry and molecular Genetics, Rutgers New Jersey Medical School, Newark, New Jersey, United States of America; 2 The Gedroic Center, Morristown, NJ, United States of America; 3 Meridian Health, Jersey Shore University Medical Center, Neptune, NJ, United States of America; University of Texas at San Antonio, UNITED STATES

## Abstract

**Background:**

Tick-borne infections have been increasing steadily over the years, with co-infections with *Borrelia burgdorferi* and *Babesia microti/divergens* emerging as a serious health problem. *B*. *burgdorferi* is a spirochetal bacterium that causes Lyme disease while protozoan pathogens belonging to *Babesia* species are responsible for babesiosis. Currently used serological tests do not always detect acute Lyme disease or babesiosis, and fail to differentiate cured patients from those who get re-infected. This is a major problem for proper diagnosis particularly in regions endemic for tick-borne diseases. Microscopy based evaluation of babesiosis is confirmatory but is labor intensive and insensitive such that many asymptomatic patients remain undetected and donate blood resulting in transfusion transmitted babesiosis.

**Results:**

We conducted multiplex qPCR for simultaneous diagnosis of active Lyme disease and babesiosis in 192 blood samples collected from a region endemic for both diseases. We document qPCR results obtained from testing of each sample three times to detect infection with each pathogen separately or together. Results for Lyme disease by qPCR were also compared with serological tests currently used for Lyme disease when available. Considering at least two out of three test results for consistency, 18.2% of patients tested positive for Lyme disease, 18.7% for co-infection with *B*. *burgdorferi* and *B*. *microti* and 6.3% showed only babesiosis.

**Conclusions:**

With an 80% sensitivity for detection of Lyme disease, and ability to detect co-infection with *B*. *microti*, multiplex qPCR can be employed for diagnosis of these diseases to start appropriate treatment in a timely manner.

## Introduction

Tick-borne infections have shown an alarming increase in the last decade. According to the CDC, approximately 300,000 cases of Lyme disease and 2,000 cases of babesiosis occur in the United States per year [1, 2]. Lyme disease caused by the spirochete group *Borrelia burgdorferi* sensu lato is also prevalent in Europe. *Ixodes scapularis* ticks transmit several pathogens including *B*. *burgdorferi* and *Babesia microti*. In the U.S.A, Lyme disease is endemic in the Northeastern and upper Midwestern states with some cases also reported in northern California, Oregon, and Washington [2]. *Babesia* species are protozoan parasites that cause malaria like febrile illness with most cases of babesiosis attributed to *B*. *microti*, while some patients are also infected by *B*. *duncani* in Western states [3, 4]. *B*. *burgdorferi* is an extracellular, highly adherent pathogen while *Babesia* species are intracellular parasites of erythrocytes. *B*. *burgdorferi* and *B*. *microti* are the most common tick-borne co-infections in the Eastern United States accounting for 40–80% of concurrent infections in different years [5, 6]. Recently, Diuk-Wasser summarized outcomes of several studies, which demonstrated that up to 40% of Lyme disease patients are also infected with *B*. *microti*, and 2/3^rd^ of babesiosis patients were also infected with *B*. *burgdorferi* in the Northeastern USA [6]. These results emphasize the importance of development and testing of a more efficient and high throughput assays to examine *B*. *burgdorferi* and *B*. *microti* simultaneously among patients particularly in the endemic regions of North America and Europe.

Upon transmission of *B*. *burgdorferi* by ticks, Lyme disease usually starts with non-specific flu like manifestations. Lyme spirochetes disseminate to various organs including the heart, joints and central nervous system using the blood as a conduit, and colonize different tissues resulting in systemic disease. Inability to diagnose and treat Lyme disease early in infection can lead to severe symptoms that can persist long after the conclusion of antibiotic treatment [7–12]. Patients suffering from this post-treatment Lyme syndrome show subjective manifestations such as chronic fatigue, musculoskeletal pain and malaise, memory loss, and inability to concentrate, all of which significantly reduce the patient’s quality of life [10]. Therefore, it is important to detect *B*. *burgdorferi* during the early stages of infection to treat the disease successfully.

Currently available FDA approved serological tests for *B*. *burgdorferi* infection cannot be used to detect acute infection before the adaptive immune response is induced [13]. The positive predictive value (PPV) of the two-step testing procedure approved by the FDA depends both on the prevalence of the disease in the region, and on the sensitivity and specificity of the commercial kit used [14–16]. Overall, the sensitivity of serological tests for the diagnosis of Lyme disease is reported to vary between 50–97%, and is highly dependent on the stage and disease manifestations of the patient [17]. In addition, antibodies to *B*. *burgdorferi* persist even after spirochete clearance, making it difficult to ascertain when the disease has been cured. Variation in the adaptive immunological response to different strains may also affect the sensitivity of the test. Furthermore, re-infection cannot be determined using these tests, a serious problem in the endemic regions. Therefore, there is a desperate need for a technically simple, rapid, and accurate test that can be readily automated to unequivocally diagnose active, acute, and post-treatment Lyme disease.

*Babesia* species are harbored by the same reservoir host, white footed mouse, as *B*. *burgdorferi* and can also be transmitted by blacklegged ticks. Indeed, co-infections of *Ixodes* species ticks with *B*. *burgdorferi* and *B*. *microti* and of the reservoir hosts have been increasing steadily over the years [18–22], and have resulted in an associated increase of co-infected individuals [5, 6, 23, 24]. Babesiosis manifestations in humans can range from asymptomatic in immunocompetent hosts to life threatening in immunocompromised, splenectomized and elderly patients [25]. Transfusion of blood containing *B*. *microti* infected erythrocytes from asymptomatic patients can result in transfusion-transmitted babesiosis (TTB) [26, 27]. Previous studies have shown that patients co-infected with *B*. *burgdorferi* and *B*. *microti* display more extensive symptoms that persist longer and also exhibit more severe disease than in Lyme disease patients [28–30]. Additionally, antibiotics used for treatment of Lyme disease are ineffective against babesiosis, further complicating the treatment of co-infections. Thus, accurate early diagnosis of co-infected individuals is imperative to provide the most effective treatment, and decrease the chance of long-lasting deleterious effects.

Diagnosis of both *B*. *microti* and *B*. *burgdorferi* infection in patients is subject to many challenges. One method used is the microscopic evaluation of Giemsa-stained blood smears. Though confirmatory, this is a time-consuming method that requires specific expertise, which limits its use for large-scale testing. This method is also limited in its application since intermittent or low levels of parasitemia are difficult to detect. Specific antibody-dependent methods can also be used for diagnosis of babesiosis, but the typical problems associated with serological tests also apply to their use for babesiosis diagnosis. Several laboratories have started using more sensitive and cost-effective PCR tests for diagnosis of babesiosis in both Europe and the United States and have found that real-time PCR assays are highly sensitive for detection of infection by *Babesia* spp. in patients [31–45]. We report here that our published real-time PCR assay [46] is also effective in diagnosing patients infected with *B*. *burgdorferi*.

In this report, we describe an accurate, sensitive multiplex quantitative PCR (qPCR) assay, that uses specific molecular beacon probes [46] to detect the presence *B*. *burgdorferi* and *B*. *microti* DNA in patient samples. This method detected a higher percentage of co-infected samples than were diagnosed using other methods. Thus, our qPCR assay can be effective for diagnosis of Lyme disease and babesiosis in patients even at the acute stage, whether the causative agents are present separately or together. This multiplex assay would be especially useful in the Northeastern and Upper Midwestern states, which are endemic for both pathogens [1, 2] as well as for the endemic regions of Europe for tick-borne infections. A qPCR-base test also could provide invaluable information about infected blood donated by patients asymptomatic for babesiosis.

## Materials and methods

### Human samples

During a visit to either Gedroic Center or the Jersey Shore University Medical Center (JSUMC), physicians ordered blood samples collection from 192 patients from three different counties in New Jersey for blood tests, including that for various infections [45]. Remaining unused aliquots of samples after clinical testing were provided to Dr. Parveen’s laboratory for this study. Physicians ordered blood collection only requires verbal, and not written, consent from patients. Patients presenting with different clinical symptoms were recommended for testing for tick borne diseases for initial evaluation or follow-up care. At the Gedroic Center, a history of tick bite, and patients presenting with high fever (>102 ^o^F) eight weeks after noticing a tick-bite were suspected of suffering from babesiosis. Furthermore, if a patient reported a history of erythema migrans indicating potential tick-borne infection, or exhibited two of the three symptoms, night sweats, shortness of breath and frontal headaches, a high index of *Babesia* infection was considered and samples were sent to IGeneX. *B*. *microti* presence on the air-dried blood smears on slides was examined by Fluorescence in situ hybridization (FISH) using 18S rDNA/rRNA target. Blood samples from patients, who reported a tick bite, or presented with migratory joint pain, mild to moderate fatigue, mental confusion/cognitive dysfunction and occipital headaches, were sent for serological testing for Lyme disease. Testing at Stony Brook Laboratory was conducted using two-step serological test for Lyme disease. Blood samples from 106 patients were collected at the Gedroic Center that also included patients who were considered asymptomatic for babesiosis and Lyme disease and were not sent for testing for tick-borne diseases.

Patient identification criteria for testing at JSUMC for babesiosis included either a history of exposure to a tick bite or patients who did not recall a tick bite but had: fever, plus or minus rash; malaise, fatigue, joint pain; anemia, with or without neutrophilia, and decrease platelet counts. Blood testing for babesiosis was conducted by microscopic examination of Giemsa-stained smears at JSUMC. Blood samples from patients with either a tick-bite and erythema migrans, or those having fever, arthralgia; malaise, fatigue and headaches when tick-bite history, were tested using Zeus Lyme VIsE1/pepC10 IgM/IgG antibodies at JSUMC. Among 86 samples collected at Meridian hospitals, samples not tested for either Lyme disease or babesiosis were also included. Some of these served as healthy cohorts when neither the results for tick-borne diseases were positive nor symptoms associated with these diseases were observed. Thus, results from a total of 192 samples conducted by clinics at two different locations are presented in this study.

### Ethics statement

Patients were not recruited for this study. Blood samples collection was ordered by physicians at two locations for blood analyses and/or for testing for various infections during patients’ visit to the clinic/hospital. Therefore, the Institutional review board (IRB) approved protocol or patients consent were not needed for blood collection. Blood collected in Ethylenediaminetetraacetic acid (EDTA) containing tubes was sent to clinical laboratories for testing for infectious diseases. After retrieval of aliquots to send to the testing laboratories, the remaining samples were provided to conduct this study at Parveen’s laboratory at the New Jersey Medical School (NJMS). Samples were provided in a coded, de-identified manner to preserve patient anonymity. Our qPCR experiments with human blood were conducted under the exempt IRB protocol of Dr. Parveen at NJMS, in accordance with the ethical standards of the responsible committee on human experimentation, of Rutgers-NJMS, and in agreement with the Helsinki Declaration of the World Medical Association. Department of Health and Human Services Federal Wide Assurance is provided to NJMS for work involving human samples.

### *B*. *burgdorferi* and *B*. *microti* quantitation by qPCR

Multiplex qPCR was conducted using primers and molecular beacon probes for *recA* amplicon for *B*. *burgdorferi* and *Bmtpk* amplicon for *B*. *microti* amplification and detection as previously described [46]. We used whole blood from each patient for isolation of DNA and determined the presence of both pathogens by qPCR using the respective standard curves [45]. The presence of leukocytes in whole blood allowed us to include human *actA1*amplicon as an internal control to ensure that DNA quality is suitable for PCR.

### Lyme C6 enzyme-linked immunosorbent assay (ELISA)

Lyme C6 ELISA was performed and data analyzed per manufacturer’s instructions (Immunetics: *B*. *burgdorferi* (Lyme) ELISA kit: DK-E352-096) using patient plasma samples diluted at 1:50. The assay calibrators were used to calculate the Lyme index for each patient sample, which directly classifies each sample as positive, negative or equivocal for *B*. *burgdorferi* infection. Lyme Index was determined according to manufacturer’s guidelines. Briefly, A_450_ of ≤0.90 was considered negative, 0.91–1.09 equivocal and ≥1.10 as positive result in the assay.

### Statistical analysis

We determined the sensitivity of qPCR as a measure of the proportion of subjects that tested positive for *B*. *burgdorferi* infection as compared to the disease state of the subject based upon clinicians’ determination and according to tests conducted by clinical laboratories and our laboratory in this study, i.e., two-tier serological tests and C6 ELISA for Lyme disease. Specificity measure in our study indicated the proportion of subjects that tested negative by qPCR given that the subjects did not have Lyme disease by these two types of serological tests. Detailed analysis of data and formulae used for various parameters is shown in results section.

## Results

### Testing of patient samples by two-tier serological tests and C6 ELISA

Of the 192 patient samples, 36 were asymptomatic for Lyme disease, while 156 presented with symptoms consistent with possible tick-borne infection likely due to *B*. *burgdorferi*. Blood samples from all symptomatic patients were sent to diagnostic laboratories to be tested for Lyme disease by the CDC recommended two-tier serological tests. After testing samples by either enzyme-linked immunosorbent assay (ELISA) or indirect immunofluorescence assay (IFA) as primary tests, samples were further tested by the standardized Western blotting method to detect antibodies against specific *B*. *burgdorferi* protein markers. Forty-nine of the 156 samples (31%) tested positive for Lyme disease by these two-tier serological tests, while 107 samples (69%) tested negative.

**Fig 1:**
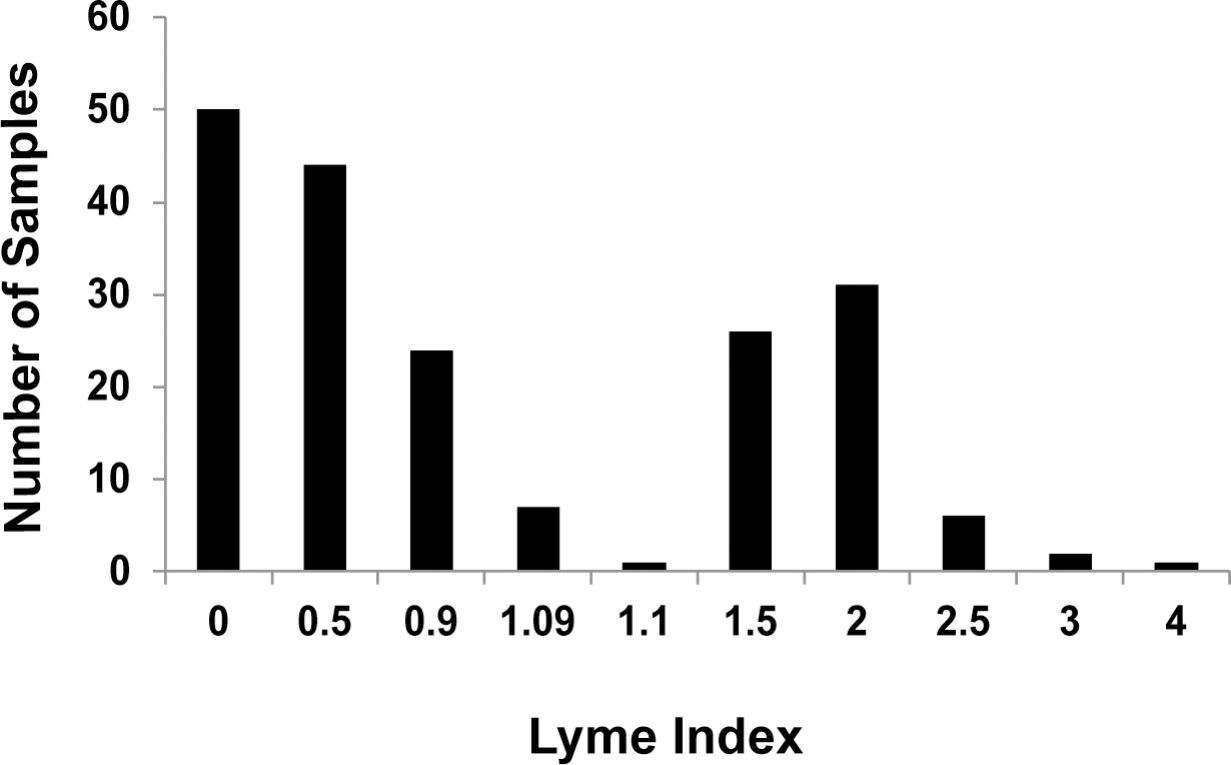
Diagnosis of *B*. *burgdorferi* infection by C6 Lyme ELISA. Histogram showing the Lyme Index distribution produced by C6 Lyme ELISA conducted on all 192 patient samples. An index ≤ 0.90 is a negative Lyme diagnosis (118 samples), an index of 0.91–1.09 is an equivocal result (7 samples), and an index ≥ 1.10 is a positive Lyme diagnosis (67 samples).

C6 Lyme ELISA uses a synthetic conserved peptide (C6 peptide) derived from the antigenic VlsE protein of *B*. *burgdorferi*, and has been reported to be more sensitive for diagnosis of Lyme disease compared to the recommended two-tier testing for patients in North America [15, 47–49]. Testing of all 192 samples detected 67 (35%) to be positive for antibodies against *B*. *burgdorferi*, while 118 (61%) tested negative (Fig 1). Seven samples (4%) produced an equivocal result. Of the 156 symptomatic patient samples tested by two-tier serology, 49 samples (31%) tested positive for Lyme disease. Thus, C6 serology corroborates Lyme disease diagnosis by two-tier serology. Combined results from all serological tests with the respective samples are documented in S1 Table.

### Comparison of qPCR assay for Lyme disease with serological test results

Patient samples were tested for tick-borne infections either at JSUMC or through commercial laboratories by Gedroic Center. To determine the efficacy of our previously optimized qPCR assay for diagnosis of Lyme disease in patient samples [46], we compared the qPCR results with two-tier serological test results provided by Drs. Gedroic and Rojtman. We found that 114 of 156 samples tested were positive by qPCR at least once and PCR cycle number for detection was below 40 (Table 1 and S1 Table). Nine samples positive by two-tier serological tests and negative by qPCR indicate the likely absence of spirochetes in blood at the time of sample collection. This is not surprising because *B*. *burgdorferi* is known to present only transiently in blood during acute phase of infection. We also cannot rule out the possibility that the level of spirochetes was below the level of detection of qPCR. In fact, a significant number of samples tested positive only once among three repeats of the test out of 146 qPCR positive samples indicating that the presence of spirochetes in blood is often at the lower end of detection limit of qPCR. We also used C6 ELISA as a complementary test for diagnosis of patient samples for Lyme disease to resolve this problem. These results also emphasize the caveat that an efficient detection of *B*. *burgdorgferi* in blood is dependent on collection of sample during active dissemination phase of infection.

**Table 1:** Diagnosis of Lyme disease by qPCR and two-tier serology.

qPCR	Two-tier Serological test results		Total
	Positive	Negative	
Positive (at least 1/3 repeats)	40	74	114
Negative	9	33	42
Total	49	107	156
**qPCR metrics**		**Standard Error**	**95%** ^a^**CI** **(Lower, Upper Bound)**
Sensitivity	81.6%	5.5%	(70.79%, 92.47%)
Specificity	30.8%	4.5%	(22.09%, 39.59%)
PPV	35.1%	4.5%	(26.33%, 43.85%)
NPV	78.6%	6.3%	(66.16%, 90.98%)

^a^CI: Confidence Interval

#### Data analysis for Table 1

$$\mathrm{Sensitivity}=\frac{\#\;\mathrm{of}\;\mathrm{True}\;\mathrm{Positives}}{\#\;\mathrm{with}\;\mathrm{Disease}\;}=\frac{40\;}{49\;}=0.8163=81.63\%$$

$$\mathrm{Standard}\;\mathrm{Error}\;\mathrm{of}\;\mathrm{Sensitivity}=\sqrt{\frac{Sensitivity*(1-Sensitivity)}{\#\;\mathrm{with}\;\mathrm{Disease}}}=\sqrt{\frac{0.816*(1-0.816)}{49}}=0.0553=5.53\%$$

$$\mathrm{Specificity}=\frac{\#\;\mathrm{of}\;\mathrm{True}\;\mathrm{Negatives}}{\#\;\mathrm{without}\;\mathrm{Disease}\;}=\frac{33\;}{107\;}=0.3084=30.84\%$$

$$\mathrm{Standard}\;\mathrm{Error}\;\mathrm{of}\;\mathrm{Specificity}=\sqrt{\frac{Specificity*(1-Specificity)}{\#\;\mathrm{without}\;\mathrm{Disease}}}=\sqrt{\frac{0.3084*(1-0.3084)}{107}}=0.0446=4.46\%$$

$$\mathrm{PPV}=\frac{\#\;\mathrm{of}\;\mathrm{True}\;\mathrm{Positives}}{\#\;\mathrm{of}\;\mathrm{Positive}\;\mathrm{Calls}\;}=\frac{40\;}{114\;}=0.3509=35.09\%$$

$$\mathrm{Standard}\;\mathrm{Error}\;\mathrm{of}\;\mathrm{PPV}=\sqrt{\frac{PPV*(1-PPV)}{\#\;\mathrm{of}\;\mathrm{Positive}\;\mathrm{Calls}}}=\sqrt{\frac{0.3509*(1-0.3509)}{114}}=0.0447=4.47\%$$

$$\mathrm{NPV}=\frac{\#\;\mathrm{of}\;\mathrm{True}\;\mathrm{Negatives}}{\#\;\mathrm{of}\;\mathrm{Negative}\;\mathrm{Calls}\;}=\frac{33\;}{42\;}=0.7857=78.57\%$$

$$\mathrm{Standard}\;\mathrm{Error}\;\mathrm{of}\;\mathrm{NPV}=\sqrt{\frac{NPV*(1-NPV)}{\#\;\mathrm{of}\;\mathrm{Negative}\;\mathrm{Calls}}}=\sqrt{\frac{0.7875*(1-0.7875)}{42}}=0.0633=6.3$$

Sensitivity of qPCR was approximately 82% and Negative Predictive Value (NPV) 79%. The low specificity and Positive Predictive Value (PPV) observed are attributable to the unavailability of a Gold standard test for Lyme disease for comparison and due to the inability of serological tests to detect acute disease in a significant number of samples tested.

For further analysis of our qPCR, we compared results with total output from all serological tests including C6 ELISA. We found that 146 samples (76%) were positive in at least one of the three qPCR repeats conducted for each sample, such that sensitivity of the assay was approximately 80%, and specificity 29.1% when compared with all serological tests (Table 2 and S1 Table). Thus, sensitivity and specificity of qPCR compared to 2-tier serological tests remained almost unchanged when we included results from C6 ELISA. Again, somewhat lower PPV for qPCR is because the serological tests, which are used for comparison here, are not the Gold standard tests particularly for acute Lyme disease.

**Table 2:** Diagnosis o Lyme disease by qPCR and serological tests.

**qPCR**	**Serological test results**		**Total**
	**Positive**	**Negative**	
Positive (at least 1/3 repeats)	83	61	144
Negative	21	25	46
Total	104	86	190
**qPCR metrics**		**Standard Error**	**95%** ^a^**CI** **(Lower, Upper Bound)**
Sensitivity	79.8%	3.9%	(72.1%, 87.5%)
Specificity	29.1%	4.9%	(19.5%, 38.7%)
PPV	57.6%	4.1%	(49.6%, 65.7%)
NPV	54.4%	7.3%	(40%, 68.7%)

^a^CI: Confidence Interval. Two samples gave equivocal results by C6 ELISA and were not tested by 2-tier serological tests

Some samples (21 in total), were serologically positive but negative by qPCR (Table 2), suggesting that patients had persistent antibodies after clearance of infecting spirochetes and thus, affect NPV values. Alternatively, due to transient presence of Lyme spirochetes in blood, *B*. *burgdorferi* were either absent in blood at the time of samples collection or their numbers were below the detection limit of qPCR. Forty-six serologically positive samples were positive by qPCR only once; indicating that these samples possibly had spirochete numbers at, or close to the lowest detection limit of qPCR.

**Fig 2:**
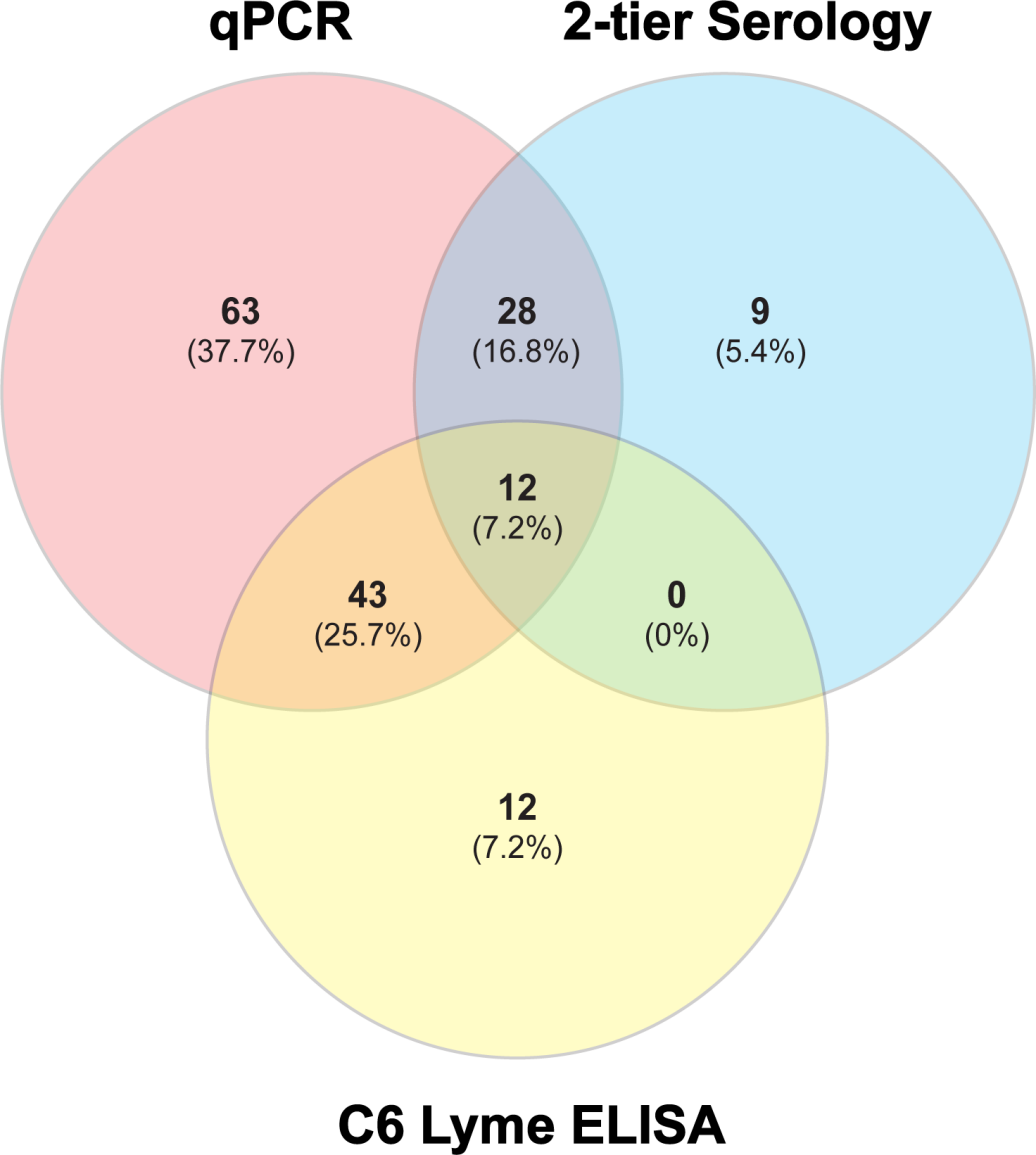
Venn diagram summarizing the agreement in positive diagnoses of Lyme disease using different diagnostic methods. *B*. *burgdorferi* infection can be detected by qPCR at a higher rate than C6 Lyme ELISA and 2-tier serological tests. There is significant overlap among all three tests.

Positive test results for Lyme disease are further summarized in the form of Venn diagram (Fig 2) for ease of comparison, with 67 samples (40.1%) positive by C6 ELISA, 49 samples (29.4%) positive by two-tier serological tests and highest, 146 samples (76%) positive by qPCR. Thus, 37.7% samples were positive only by qPCR compared to 7.2% and 5.4% samples that tested positive only by C6 ELISA or 2-tier serological tests, respectively. Samples that showed positive results by serological tests and negative by qPCR may depict either a past infection detectable by antibodies despite spirochete clearance or that patients were on the verge of cure of Lyme disease. In all these situations, there is likelihood of no to low *B*. *burgdorferi* presence in blood that could be below the detection limit of qPCR. The lack of overlap between 92 positive samples by either two-tier tests or C6 ELISA was surprising and highlights the problem associated with the use of different kits/tests for serological evaluation and the absence of ideal standards for diagnosis of Lyme disease. Two-tier serological test positive results for 24% samples also showed positive reaction with qPCR. A higher agreement was obtained between samples positive by C6 ELISA and qPCR with 32.9% samples positive by both tests.

### Testing of samples for babesiosis

As we reported previously [45], clinical samples were also tested either by IFA, FISH or microscopic examination of Giemsa-stained blood smears for the presence of *B*. *microti* (results are summarized here in S1 and S2 Tables). Based upon the lack of symptomatology, 131 samples were not tested by the clinical laboratories using any test for *B*. *microti*. Our qPCR testing of these samples showed that 38% were positive for *Babesia* presence [45]. A high congruency of DNA based assays, qPCR and FISH was observed because 27 of 28 FISH positive samples (96%) were also positive by qPCR. Interestingly, of 78 samples untested through Gedroic Center due to the absence of symptoms, 22 were positive by our qPCR. Direct detection of *Babesia* by labor-intensive microscopic examination of patient blood smears was used only for a few J samples. By considering individuals positive for *Babesia* infection when results from currently available microscopy, FISH or serological tests were positive, we found that our qPCR is highly sensitive (96.4%) and showed a specificity of 70.5% for *Babesia* presence [45]. Somewhat lower specificity of qPCR could be because serological tests may not depict active infection in all cases. Samples without a known history of tick bite and negative by all tests for both Lyme disease and babesiosis served as healthy, uninfected cohorts for tick-borne diseases here.

### Detection of *B*. *burgdorferi-B*. *microti* co-infections by qPCR

Assessing the efficacy of our qPCR as a multiplex assay for detection of co-infections with *B*. *burgdorferi* and *B*. *microti* showed it to be very effective (Table 3 and S1 Table). Our results show co-infection rate of approximately 39% among our 192 patient samples, while single infection with *B*. *microti* was almost 10%. We found that qPCR could uniquely detect the presence of both infections simultaneously, which is not yet possible by other standard diagnostic tests. Furthermore, it is possible to detect active infection by both tick-borne pathogens using multiplex qPCR particularly when bacteremia/parasitemia is low and patients are asymptomatic for babesiosis as commonly observed in healthy but *B*. *microti* infected individuals [Table 3, S1 Table and [45]].

**Table 3:** Detection of single and co-infections of *B*. *burgdorferi* and *B*. *microti* using qPCR.

	Uninfected	Single infection		Co-infection
		*B*. *burgdorferi*	*B*. *microti*	
qPCR ++ or +++^a^	0	35 (18.2%)	12 (6.3%)	36 (18.7%)
qPCR +^b^	0	37 (19.3%)	6 (3.1%)	38 (19.8%)
qPCR Negative	28 (14.6%)	0	0	0
Total: **192**	28 (14.6%)	72 (37.5%)	18 (9.4%)	74 (38.5%)

^a^qPCR ++ or +++: Samples positive two or three times of the 3 qPCR assays done

^b^qPCR +: Samples positive one of the 3 qPCR assays done

## Discussion

Symptoms-based diagnosis of various tick-borne diseases is problematic due to similar early clinical manifestations. Although it is possible to use serological tests in a high throughput manner during the post-acute phase of different infectious diseases, problems associated with these tests are well-established and hinder diagnosis of active *B*. *burgdorferi* infection using FDA approved two-tier serological tests [50, 51]. Subjective interpretation of Western blot results as the second-tier test further diminishes accuracy with an average of 70–80% serological test efficiency noted for diagnosis of Lyme disease. However, accuracy of a single C6 ELISA test sensitivity is reported to be slightly higher for Lyme disease than the two-tier serological test [49]. PCR-based assays have had only a limited level of success for Lyme disease diagnosis due to the presence of small numbers of spirochetes often transiently present in blood, and because of the design imperfections [52–54]. Our multiplex qPCR, with a much improved design that use of molecular beacon probes tagged with different fluorophores was highly sensitive and specific in detection of both *B*. *burgdorferi* and *B*. *microti* spiked blood [46].

We used this qPCR assay for retrospective diagnosis of Lyme disease and babesiosis in patient samples, collected from a region endemic for tick-borne diseases during transmission season. Our results highlight the advantages of qPCR-based diagnosis of Lyme disease. This method is independent of an immune response to infection, and is able to detect acute infection. In the absence of confirmatory tests available for Lyme disease, the PPV of the qPCR was relatively low though still above average, 58% when we compared it with all serological test results combined, suggesting that many serologically negative samples that showed positive results by qPCR are likely from patients with acute disease who lack antibodies against *B*. *burgdorferi* antigens. Although we cannot rule out false negative detection by qPCR, an NPV of 54.4% suggests that serologically positive samples that were negative by qPCR (Fig 2 and S1 Table) could be attributable to persistent antibodies in some patient samples, even though these patients no longer have Lyme disease or to the absence/low levels of spirochetes in blood below the detection limit of qPCR at the time of sample collection.

A major advantage of our multiplex qPCR is its ability to detect different tick-borne infections simultaneously. Indeed, we were able to detect a surprisingly high rate of *B*. *burgdorferi-B*. *microti* co-infections (~39%) in patient samples collected from a state endemic for tick-borne diseases. These results could be attributable to a high rate of infections with both pathogens in the counties where this study was undertaken. Antibiotic treatment used for Lyme disease is ineffective for babesiosis, which is often asymptomatic in immunocompetent individuals. Patients with severe babesiosis need hospitalization and the disease can even cause death of such patients due to multi-organ failure [55]. Therefore, diagnosis by a qPCR method gives the advantage of timely and appropriate treatment of patients and thus, could help in the prevention of TTB. Serum contains nucleases such that pathogenic nucleic acids do not persist long in the host after the disease has been cured. This allows for the use of nucleic acids-based assays as a test of cure for a variety of infectious diseases [31]. In fact, in addition to our studies, a comparative analysis conducted by the CDC last year further emphasized the importance of molecular beacon probes-based qPCR tests for babesiosis [44, 45], Success of such assays in other well-known laboratories highlights the significance of our test. Multiplex assays have not been employed yet by any other laboratory for tick-borne diseases further emphasizing the importance of our assay in the simultaneous detection of tick-borne pathogens in endemic regions.

## Conclusions

Our multiplex qPCR offers a unique advantage for simultaneous diagnosis of Lyme disease and babesiosis in co-infected patients. Additionally, our qPCR can be used to efficiently detect *B*. *microti* presence in donated blood.

## Supporting information

S1 Table Master table for patients test data. (PDF)

S2 Table Summary of results of all tests used for Lyme disease and babesiosis. (PDF)

## Abbreviations

qPCR: Quantitative PCR
TTB: transfusion-transmitted babesiosis
ELISA: Enzyme Linked Immunosorbent Assay
FISH: Fluorescence in situ hybridization
NPV: Negative Predictive Value
PPV: Positive Predictive Value
