# Supplementary material for: Efficient detection of symptomatic and asymptomatic patient samples for Babesia microti and Borrelia burgdorferi infection by multiplex qPCR
Source: PLoS One. 2018 May 10;13(5):e0196748. doi: 10.1371/journal.pone.0196748 (PMC5945202; doi:10.1371/journal.pone.0196748)
Supplement: S1 Table — (PDF) [file pone.0196748.s001.pdf]

| Supplementary Table S1. Master Table | | | | |
|---|---|---|---|---|
| | *B. Burgdorferi* | | *B. microti* | |
| Sample (Total number of samples) | qPCR* | Serology[†] | qPCR* | Microscopy/FISH |
| J32, J66, J75, KG44, KG62, KG64, KG65, KG87 (8) | + | + | - | - |
| J03, J04, J05, J07, J12, J30, J45, J47, J48, J52, J54, J59, J62, J63, J65, J68, J69, J70, J71, J73, J76, J77, J84, KG15, KG18, KG21, KG22, KG26, KG28, KG33, KG35, KG59, KG67, KG83, KG88, KG89, KG90, KG91, KG92, KG102 (40) | + | + | + | NT |
| KG82 (1) | + | +/- | - | NT |
| J38, J46, KG42, KG70, KG71, KG73, KG77 (7) | + | - | - | - |
| J06, J09, J21, J79, J83, KG19, KG25, KG30, KG50, KG52, KG55, KG61, KG76, KG81, KG101 (15) | + | - | - | NT |
| J50, KG39 (2) | - | + | - | - |
| J28, J33, J44, J60, KG20, KG38, KG47, KG51, KG58 (9) | - | + | - | NT |
| **Total - *B. burgdorferi* single infection: 82** | | | | |
| J17, KG27, KG57 (3) | - | - | + | + |
| J15, J25, KG41, KG43 (4) | - | - | + | NT |
| KG49 (1) | - | - | + | - |
| KG04 (1) | - | - | - | + |
| **Total - *B. microti* single infection: 9** | | | | |
| J23, J35, J56, J78, KG07, KG13, KG16, KG53, KG54, KG63, KG85, KG93, KG98, KG100 (14) | + | + | + | + |
| J41, KG68, KG86 (3) | + | + | + | - |
| J22, J36, J58, J64, J67, J72, J74, J80, J82, J85, J87, KG02, KG08, KG09, KG11, KG29, KG84, KG97 (18) | + | + | + | NT |
| J01, J02, J08, J19, J20, J31, J51, J57, J86, KG05, KG10, KG60, KG78, KG95 (14) | + | - | + | + |
| KG45, KG48, KG72, KG99, KG103 (5) | + | - | + | - |
| J10, J11, J13, J29, J37, J42, J55, J81, KG01, KG03, KG06, KG24, KG36, KG56, KG94, KG96, KG104, KG105, KG106 (19) | + | - | + | NT |
| KG69 (1) | + | - | - | + |
| KG75 (1) | + | +/- | + | NT |

| | | | | |
|---|---|---|---|---|
| J18, J40, J53, KG17 (4) | - | + | + | + |
| J24, J39, J43, J49, KG12, KG46 (6) | - | + | + | NT |
| **Total - co-infected: 85** | | | | |
| KG37, KG66, KG74, KG79 (4) | - | - | - | - |
| J14, J16, J26, J27, J34, KG14, KG23, KG31, KG32, KG34, KG40, KG80 (12) | - | - | - | NT |
| **Total - uninfected: 16** | | | | |
| **Total samples: 192** | | | | |
| *:+ for qPCR denotes +, ++ & +++ Ϯ: +/- is an equivocal result, NT: samples not tested | | | | |
