# Supplementary material for: Efficient detection of symptomatic and asymptomatic patient samples for Babesia microti and Borrelia burgdorferi infection by multiplex qPCR
Source: PLoS One. 2018 May 10;13(5):e0196748. doi: 10.1371/journal.pone.0196748 (PMC5945202; doi:10.1371/journal.pone.0196748)
Supplement: S2 Table — (PDF) [file pone.0196748.s002.pdf]

Supplementary Table S2. Summary of results for different diagnostic tests for Lyme disease and babesiosis

Samples Description	Number of Samples for Lyme disease				Number of Samples for babesiosis						qPCR Positive		
	Positive		Negative by serology	Total Tested£	Microscopy		FISH		IFA≠		Lyme	Babesiosis	Both
	2-Tier	C6 ELISA*			Positive	Negative	Positive	Negative	Positive	Negative			
Positive qPCR KG samples from Gedroic Center (Morris County, NJ)	10	33+5*	37	**70**Δ	NT	NT	18	15	0	1	39	8	41
Negative qPCR KG samples from Gedroic Center (Morris County, NJ)	1	8	17	**26**	NT	NT	2	15	0	3	N/A	N/A	N/A
Positive qPCR J samples from JSUMC (Ocean & Monmouth Counties, NJ)	30	22+1*	24	**74**€	17	1	NT	NT	7	20	33	10	33
Negative qPCR J samples from JSUMC (Ocean & Monmouth Counties, NJ)	8	4+1*	8	**18**	0	6	NT	NT	1	37	N/A	N/A	N/A
**Total**	**49**	**67**	**86**	**190**	**17**	**7**	**20**	**30**	**8**	**61**	**72**	**18**	**74**

NT: Not Tested
N/A: Not Applicable
*: Seven samples gave an equivocal result by C6 ELISA
£: Two samples were equivocal by C6 serology and not tested by 2-tier serology. These are not listed in Lyme disease samples.
Δ: Ten samples were positive by both C6 ELISA and 2-tier serology
€: Two samples were positive by both C6 ELISA and 2-tier serology
≠: IFA was used as an alternative test for J samples. Only four KG samples were tested by IFA for babesiosis
